# Malaria and risk of lymphoid neoplasms and other cancer: a nationwide population-based cohort study

**DOI:** 10.1186/s12916-020-01759-8

**Published:** 2020-10-30

**Authors:** Katja Wyss, Fredrik Granath, Andreas Wångdahl, Therese Djärv, Michael Fored, Pontus Naucler, Anna Färnert

**Affiliations:** 1 Division of Infectious Diseases, Department of Medicine Solna, Karolinska Institutet, Stockholm, Sweden; 2 Department of Infectious Diseases, Karolinska University Hospital, 171 76 Stockholm, Sweden; 3 Clinical Epidemiology Division, Department of Medicine Solna, Karolinska Institutet, Stockholm, Sweden; 4 Department of Infectious Diseases, Västerås Hospital, Västerås, Sweden; 5 Division of Clinical Medicine, Department of Medicine Solna, Karolinska Institutet, Stockholm, Sweden; 6 Function of Emergency Medicine, Karolinska University Hospital, Stockholm, Sweden

**Keywords:** Malaria, Lymphoid neoplasms, Lymphoma, Cancer

## Abstract

**Background:**

Malaria is associated with Burkitt lymphoma among children in Sub-Saharan Africa. No longitudinal studies have assessed the long-term risk of other lymphoma or cancer overall. Here, we investigated the risk of lymphoid neoplasms and other cancer after malaria.

**Methods:**

We included 4125 patients diagnosed with malaria in Sweden in 1987–2015, identified either through the National Surveillance Database at the Public Health Agency of Sweden, the National Inpatient and Outpatient Register, or by reports from microbiology departments. A comparator cohort (*N* = 66,997) matched on sex, age and birth region was retrieved from the general population and an additional cohort with all individuals born in Sub-Saharan Africa registered in the Total Population Register in 1987–2015 (*N* = 171,756). Incident lymphomas and other cancers were identified through linkage with the Swedish Cancer Register. Hazard ratios (HRs) were assessed using Cox regression with attained age as the timescale.

**Results:**

A total of 20 lymphoid neoplasms and 202 non-haematological cancers were identified among malaria patients during a mean follow-up of 13.3 and 13.7 years, respectively. The overall risk of lymphoid neoplasms was not significantly increased (hazard ratio [HR] 1.24, 95% confidence interval [CI] 0.79–1.94), neither did we find any association with all-site non-haematological cancer (HR 0.89, 95% CI 0.77–1.02). However, in the Sub-Saharan Africa cohort, we observed an increased risk of lymphoid neoplasms after malaria diagnosis (HR 2.39, 95% CI 1.06–5.40), but no difference in the risk of other cancer (HR 1.01, 95% CI 0.70–1.45). The association could not be explained by co-infection with HIV or chronic hepatitis B or C, since the risk estimate was largely unchanged after excluding patients with these comorbidities (HR 2.63, 95% CI 1.08–6.42). The risk became more pronounced when restricting analyses to only including non-Hodgkin and Hodgkin lymphomas (HR 3.49, 95% CI 1.42–8.56).

**Conclusion:**

Individuals born in malaria-endemic areas and diagnosed with malaria in Sweden had an increased risk of lymphoid neoplasms, especially B cell lymphoma. There was no association with cancer overall nor did single malaria episodes confer an increased risk in travellers.

## Background

Nearly half of the world’s population lives at risk of malaria [1]. The acute complications of malaria are well known, but in addition to direct malaria-related morbidity and mortality, there is increasing evidence that malaria has several long-term effects. One such consequence is endemic Burkitt lymphoma, the most common cancer in children in Sub-Saharan Africa [2, 3]. The International Agency for Research on Cancer (IARC) has declared *Plasmodium falciparum* as a probable carcinogenic agent in humans [4], but besides the well-established association with Burkitt lymphoma [5–7], epidemiological evidence is limited.

Occasional epidemiological reports from endemic areas have suggested a link between malaria and non-Burkitt lymphomas in adults [8–10], as well as cervical cancer in women living in high-endemic regions of Uganda [11]. A few case-control studies from non- or low-endemic countries indicate that malaria exposure could be associated with the non-endemic (sporadic) form of Burkitt lymphoma diagnosed at adult age [12], as well as non-Burkitt non-Hodgkin lymphomas [13, 14], lymphatic malignancies [15] and nasopharyngeal carcinoma in South East Asia [16]. Most of these studies are based on self-reported history of malaria, thus prone to exposure misclassification. One nested-case control study from the UK used longitudinally collected data from primary care records to assess previous malaria exposure [12], but otherwise, there are to our knowledge no publications with longitudinal design assessing the risk of lymphomas or other cancers in adults with previous malaria. Such investigations are difficult to manage in endemic areas where reliable health and recording systems often are lacking and the degree of malaria exposure is complicated to determine due to repeated and asymptomatic infections. Here, we have taken advantage of the unique health and population registries in Sweden to study long-term effects of malaria diagnosed in a mixed population of travellers and migrants.

We performed a nationwide population-based cohort study of patients diagnosed with malaria in Sweden, with the aim to investigate if malaria is associated with an increased risk of lymphoid neoplasms and/or other cancer.

## Methods

### Setting

Residents in Sweden are issued a unique personal identification number (PIN) that is recorded in all health and census registers, enabling register linkage [17]. Malaria is a notifiable disease under the Communicable Diseases Act, and microbiologically confirmed cases (by microscopy of thick and thin blood films or, infrequently, by polymerase chain reaction) should be reported to the Public Health Agency of Sweden both by the treating clinician and the diagnosing microbiology laboratory. Malaria is generally managed by infectious disease specialists at larger hospitals, although other departments are involved in the treatment of children, pregnant women or complications requiring other specialists. Most patients diagnosed with malaria are admitted, but semi-immune individuals with mild symptoms are sometimes managed on an outpatient basis.

The Swedish National Patient Register (NPR) at the National Board of Health and Welfare includes diagnoses for an estimated 99% of all hospitalizations in Sweden, with nationwide coverage since 1987 [18]. Since 2001, specialised outpatient care also is included. The Total Population Register (TPR) at Statistics Sweden contains demographic data for all inhabitants with PIN from 1969, including sex, date and country of birth, date of death and dates and countries of immigration and emigration.

### Study population

#### Exposed cohort

**Fig. 1:**
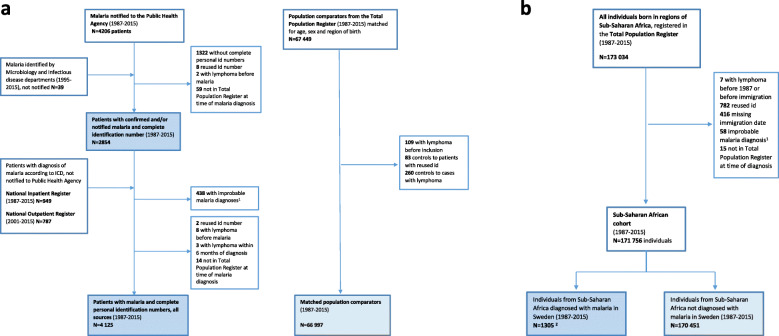
**a** Study population flow chart. ^1^Ninety-four excluded from the Inpatient Register with secondary malaria diagnoses from psychiatry, otorhinolaryngology, ophthalmology, orthopaedics, geriatrics, dermatology and surgery departments. Seventy-four excluded from the Outpatient Register with secondary malaria diagnoses from the emergency department (7), paediatric department (26) and infectious disease department (41) and 270 excluded with primary and secondary diagnoses from other departments. **b** Flow chart for the population from Sub-Saharan Africa. ^1^Twelve patients from the National Inpatient Register and 46 patients from the National Outpatient Register excluded from all analyses because of uncertain malaria diagnosis.^2^Including 1066 patients with confirmed malaria, 166 patients from the Inpatient Register and 73 patients from the Outpatient Register

Malaria patients were identified through four different sources (Fig. 1a):

1. All notified cases of malaria with complete PIN reported in the National Surveillance Database at the Public Health Agency of Sweden 1987–2015 (*N* = 2884 unique patients)
2. Additional patients with microbiologically confirmed malaria identified through hospital or laboratory records from infectious disease or microbiology departments (*N* = 39)
3. Additional patients discharged with a diagnosis of malaria, according to the International Classification of Disease ICD-10: B50.0-B54.9 or ICD-9, 084A-H, 084W and 084X, in the National Inpatient Register 1987-2015 (*N* = 949).
4. Additional patients with a diagnosis of malaria (same ICD codes as above) in the National Outpatient Register 2001–2015 (*N* = 787).

Patients from sources 1 and 2 were considered confirmed whereas cases identified via sources 3 and 4 were not necessarily microbiologically verified. For confirmed malaria diagnosed in 1995–2015, data was enriched with clinical variables from medical records, such as disease severity and *Plasmodium* species, used in previous studies [19–21].

To increase the specificity of malaria identified via the NPR, we only included the main diagnoses of malaria from the infectious diseases, paediatric and emergency medicine departments in the Outpatient Register (*N* = 344 excluded), while all patients discharged with a diagnosis of malaria were included from the Inpatient Register except those with secondary diagnoses from the departments other than infectious diseases, paediatrics, intensive care, internal medicine and obstetrics/gynaecology, and no follow-up at an infectious disease department (*N* = 94 excluded). For individuals with multiple episodes, inclusion was set at the date of the first episode with confirmed malaria.

A total of 186 patients were not registered in the TPR at the time of malaria diagnosis; however, of these, 113 had an immigration date within 1 year after diagnosis and were included from this date. Often, the actual date of immigration has occurred earlier but is set to the date of registration in Sweden (personal communication with Jonas Färnstrand, Statistics Sweden). Before becoming registered in Sweden, immigrants in contact with the health care system obtain a temporary identification number that can be connected to the PIN via electronic patient records, thereby enabling inclusion as a malaria case.

Depending on the outcome analysed, patients with either prior diagnosis of lymphoid neoplasm or all-site cancer were excluded. To minimise the possibility of misclassifying patients identified by ICD-codes in the NPR, lymphoid neoplasms and cancers diagnosed within 6 months after the malaria diagnosis were also excluded.

#### Comparator cohort

For each malaria patient, we randomly selected 20 comparators from the TPR, matched for sex, birth year (± 1 year) and region of birth categorised according to the United Nations geoscheme [22], alive and without previous malaria diagnosis on the date of malaria of the corresponding patient. Individuals with previous lymphoma or cancer were excluded.

Based on a previous study of imported malaria in Sweden, approximately 50% of patients with confirmed malaria had immigrated from countries where malaria is highly endemic [21]. In order to better control for host factors including malaria exposure and immunity, an additional cohort was assembled from the TPR composed of all individuals born in Sub-Saharan Africa, defined as the United Nations geoscheme regions Eastern Africa, Western Africa, Middle Africa and Southern Africa, who were alive and registered in Sweden at some point between 1987 and 2015 (Fig. 1b). We refer to these regions as “endemic” and all other regions as “low- or non-endemic”.

### Register linkage and follow-up

The study population was followed from the inclusion date, i.e. first episode of malaria or corresponding date for comparators, to the first diagnosis of lymphoma or cancer, emigration, death or end of study (31 January 2016), whichever occurred first. For unexposed individuals, follow-up also ended if malaria was diagnosed, and the comparator then became included as exposed. Follow-up was achieved through linkage to the following registers:

1. The Cancer Register at National Board of Health and Welfare includes the date and type of all incident cancers diagnosed in Sweden from 1958. Reporting is mandatory by both clinician and pathologist and completeness estimated to > 96 [23].
2. The TPR at Statistics of Sweden includes date and country of birth as well as migration data.
3. Cause of Death Register at National Board of Health and Welfare includes date and causes of death.

The cohort from Sub-Saharan Africa was followed from the first immigration date to Sweden, or from 1 January 1987 if immigration occurred before 1987. Individuals were censored at emigration but were allowed to re-enter the study in case of subsequent immigration episodes; thus, all registered periods of residency in Sweden were included.

### Outcome

Outcomes were defined as the first diagnosis of lymphoid neoplasms (ICD-7: 200-204) or the first diagnosis of all-site cancer excluding haematological malignancies and non-melanoma skin cancers (ICD-7: 140-190,192-199). In a subanalysis, we also assessed the risk of malignant lymphoma (non-Hodgkin, NHL and Hodgkin) using a stricter definition excluding chronic lymphatic leukaemia (CLL), myeloma, acute lymphoblastic leukaemia (ALL) and lymphoproliferative disorders. For this purpose, histopathology diagnoses referred to as SNOMED codes (Systematized Nomenclature of Medicine), available from 1993, were used for a more precise classification of the different types of lymphoid neoplasms as well as subclassification of NHL, according to InterLymph [24, 25].

### Statistics

We used Stata version 14 (StataCorp) for statistical analyses and SAS version 9.4 for data cleaning. Crude incidence rates and 95% confidence intervals (CIs) were calculated as the number of events (first diagnosis of lymphoid neoplasms, or all site non-haematological cancer during follow-up) per 1000 person-years. We estimated relative risks for malaria cases compared to the comparator cohort as hazard ratios (HR) with 95% CIs using Cox proportional hazard regression, stratified for sex, patient birth region (Sub-Saharan Africa vs non/low-endemic regions), calendar period for study entry (1987 to 1994, 1995 to 2004, 2005 to 2015) and follow-up time (< 5, 5 to 9, 10 to 19 and 20 to 30 years. Attained age was used as underlying timescale throughout the study; thus, all analyses were adjusted for age. Additional adjustments were made for sex, birth region and calendar period. The proportional hazard assumption was assessed using Schoenfeldt residuals.

Assuming that comparators born in Sub-Saharan Africa to some extent also could have been malaria exposed before arriving in Sweden, we categorised the study population into four levels of assumed increasing malaria exposure: controls with non/low-endemic origin, malaria cases with non/low-endemic origin, controls with endemic origin and cases with endemic origin, and estimated HR for lymphoma and cancer overall for all categories compared to the group of expected lowest exposure (comparator subjects with non/low-endemic origin).

For the cohort of individuals born in Sub-Saharan Africa, malaria was treated as a time-varying covariate; thus, an individual diagnosed with malaria contributed with both unexposed and exposed time. Individuals were censored at emigration, but those that re-immigrated were included as new episodes from that date. The time in between emigration and immigration was not included in risk time. However, we created a variable for estimated total attained time spent in an endemic region, based on the country of re-immigration, as a measure of previous exposure. Crude incidence rates were calculated overall and separately for sex, calendar period, follow-up time, region of birth within Africa, age at first immigration (< 18, 18 to 29, 30 to 39, ≥ 40 years), age at the end of follow-up (0 to 29, 30 to 49 and ≥ 50 years) and time in endemic region (0 to 9, 10 to 19, 20 to 29 and ≥ 30 years). Crude and adjusted HRs were stratified for total attained time in endemic region and sex.

A restricted analysis was performed excluding individuals with a diagnosis of HIV, chronic hepatitis B or C identified in the NPR (ICD 10: B20-B24, Z21, B18.0-B18.1, B18.2; ICD-9: 2795/279K, 079J, 0702-0705) before inclusion and censoring those diagnosed during follow-up at the date of HIV/hepatitis diagnosis, including up until 6 months after lymphoma diagnosis (assuming the chronic infection was present before lymphoma diagnosis).

## Results

In total, 4125 individuals diagnosed with malaria in Sweden were included in the study. Among these, 2854 patients had microbiologically confirmed malaria, of which 2815 (98.6%) notified to the Swedish Public Health Agency and 2009 (70.4%) with available clinical data. Additionally, 1271 patients with unnotified malaria were identified through malaria ICD 9/10 codes from the Inpatients Register (*N* = 841) or Outpatient Register (*N* = 430). As it was not possible to find 20 controls for all patients born outside Sweden, the total number of controls was 66,997 (mean 16 controls per patient) (Fig. 1a). This explains the difference in the proportion of patients with origin in Sub-Saharan Africa, presented among other demographic characteristics in Table 1. Other differences noted were the higher proportion of HIV and hepatitis B diagnoses among malaria cases.

**Table 1:** Characteristics of malaria patients and matched population comparators

	**Malaria patients, all sources**^**a**^ **(*N*** **= 4125)**^**b**^	**Matched comparator cohort from the general population (*N*** **= 66,997)**
**Sex**, female, *n* (%)	1622 (39.3)	26,991 (40.3)
**Age**		
Age at start of follow-up, mean (± SD) years	34.7 (± 18.5)	35.5 (± 18.9)
Attained age at end of follow-up, mean (± SD) years	48.0 (± 18.7)	49.6 (± 19.0)
Children < 18 y, at inclusion, *n* (%)	676 (16.4)	9627 (14.4)
Age ≥ 65 y, at inclusion, *n* (%)	273 (6.6)	5091 (7.6)
**Follow-up**		
Total follow-up, person-years	54,739	943,828
Average follow-up, person-years (SD)	13.3 (± 8.3)	14.1 (± 8.4)
**Country of birth**^c^		
Sub-Saharan Africa, *n* (%)	1307 (31.7)	12,758 (19.0)
Other low-endemic regions, *n* (%)	295 (7.2)	4899 (7.3)
Sweden/non-endemic region, *n* (%)	2523 (61.1)	49,332 (73.6)
**Co-infections**		
HIV before/at inclusion, or during follow-up^d^, *n* (%)	54 (1.3), 49 (1.2)	228 (0.3), 164 (0.2)^3^
Hepatitis B before/at inclusion, or during follow-up^e^, *n* (%)	26 (0.6), 39 (1.0)	235 (0.4), 307 (0.5)
Hepatitis C before/at inclusion, or during follow-up, *n* (%)	14 (0.3), 19 (0.5)	124 (0.2), 333 (0.5)
**Incident lymphoid neoplasms,** ***n*** **(%)**	**20 (0.48)**	**304 (0.45)**
Lymphoid neoplasm within 1 year, *n* (%)	0	17 (5.6)
Lymphoid neoplasm after 1–5 y, *n* (%)	5 (25.0)	65 (21.4)
Lymphoid neoplasm after > 5 y, *n* (%)	15 (75.0)	222 (73.0)
	**Malaria patients, all sources (*N*** **= 4027)**^**f**^	**Matched comparator cohort from the general population (*N*** **= 63,588)**
**Incident cancer**^**g**^**,** ***n*** **(%)**	**202 (5.0)**	**3993 (6.2)**
Total follow-up, person-years	55,361	924,517
Average follow-up, person-years (SD)	13.7 (± 8.1)	14.5 (± 8.3)

*N* total number, *n* number, *SD* standard deviation, *y* years

^a^Includes 2854 patients with confirmed malaria, of which 2815 patients notified to the Public Health Agency 1987–2015 and 39 (1.4%) cases not notified to the Public Health Agency but microbiological confirmed. Additional 1271 patients with unnotified malaria identified through the National Inpatient Register 1987–2015 (*N* = 841) and Outpatient Register 2001–2015 (*n* = 430) with a diagnosis of malaria according to ICD-10 (B50.0-B54.9) or ICD-9 (084A-H, 084W, 084X)

^b^Patients with previous lymphoid neoplasms excluded: 2 patients with notified malaria and 8 patients with malaria identified in the National Inpatient Register. Three malaria cases from the National Inpatient Register were excluded because of lymphoid neoplasm within 6 months

^c^*n* = 8 of comparators with missing information on country of birth. Two malaria cases with missing information on birth where data on birth country was retrieved through the clinical database

^d^Additionally, 1 case of HIV after lymphoma in a malaria patient and 4 among comparators

^e^Additionally, 1 case of hepatitis B after lymphoma in a malaria patient

^f^Patients with previous cancer (non-haematological) were excluded: 98 malaria patients with previous cancer and 16 with cancer within 6 months

^g^All cancers except for non-melanoma skin cancers, leukaemia and lymphoma (ICD-7: 140-190, 192-199)

### Risk of lymphoid neoplasms

The 4125 malaria patients were followed for 54,739 person-years, during which we observed 20 cases of lymphoid neoplasms, corresponding to a crude incidence of 0.37 per 1000 person-years (95% CI 0.24–0.57). Three hundred four lymphoid neoplasms were observed among the matched comparators during 943,828 person-years, corresponding to a crude incidence of 0.32 per 1000 person-years (95% CI 0.29–0.36). The majority (15/20) of lymphoid neoplasms among the malaria cases were diagnosed more than 5 years after the malaria diagnosis (Table 1).

The overall risk for incident lymphoid neoplasms among malaria patients was not increased (HR 1.24, 95% CI 0.79–1.94). There was a modest difference in the risk estimate of men and women with malaria, a tendency of higher risk in individuals born in Sub-Saharan Africa (HR 1.97, 95% CI 0.81–4.81) and inclusion after 2005 (HR 2.29, 95% CI 0.90–5.80). The overall risk was not affected by adjustment for sex, region of birth and calendar period for entry (adjusted HR [adjHR] 1.20, 95% CI 0.76–1.88) and was similar in the analysis restricted to confirmed cases only (HR 1.26, 95% CI 0.73–2.16, adjHR 1.20, 95% CI 0.69–2.07). However, for individuals of endemic origin, the association became somewhat more pronounced among confirmed cases (HR 2.32, 95% CI 0.94–5.76) (Table 2).

**Table 2:** Hazard ratios for incident lymphoid neoplasms and other cancer in malaria patients

	**Malaria, all sources**^**a**^ **(*N*** **= 4125)**			**Confirmed malaria**^**b**^ **(*N*** **= 2854)**		
	**Lymphoid neoplasms among malaria patients,** ***n*** **(%)**	**Lymphoid neoplasms among comparators,** ***n*** **(%)**	**HR (95% CI)**	**Lymphoid neoplasms among malaria patients,** ***n*** **(%)**	**Lymphoid neoplasms among comparators,** ***n*** **(%)**	**HR (95% CI)**
All	20	304	1.24 (0.79–1.94)	14	194	1.26 (0.73–2.16)
Sex						
Male	13 (65.0)	210 (69.1)	1.12 (0.64–1.95)	9 (64.2)	142 (73.2)	1.09 (0.56–2.14)
Female	7 (35.0)	94 (30.9)	1.49 (0.69–3.20)	5 (35.7)	52 (26.8)	1.68 (0.67–4.22)
Follow-up time (y)						
< 5	5 (25.0)	82 (27.0)	0.91 (0.37–2.24)	2 (14.3)	45 (23.2)	0.64 (0.16–2.65)
5–9	5 (25.0)	78 (25.7)	1.06 (0.43–2.62)	4 (28.6)	48 (24.7)	1.30 (0.57–3.60)
10–19	7 (35.0)	108 (35.5)	1.24 (0.58–2.67)	7 (50.0)	80 (41.2)	1.45 (0.67–3.14)
20–30	3 (15.0)	36 (11.8)	1.38 (0.42–4.51)	1 (7.1)	21 (10.8)	0.86 (0.12–6.43)
Years of entry						
1987–1994	9 (45.0)	109 (35.9)	1.51 (0.77–2.99)	6 (42.9)	67 (34.5)	1.69 (0.73–3.90)
1995–2004	6 (30.0)	155 (51.0)	0.73 (0.32–1.66)	5 (35.7)	103 (53.1)	0.81 (0.33–2.00)
2005–2015	5 (25.0)	40 (13.2)	2.29 (0.90–5.80)	3 (21.4)	24 (12.4)	1.90 (0.57–6.33)
Region of birth						
Sub-Saharan Africa	6 (30.0)	25 (8.2)	1.97 (0.81–4.81)	6 (42.9)	21 (10.8)	2.32 (0.94–5.76)
Non/low-endemic	14 (70.0)	279 (91.8)	1.05 (0.61–1.80)	8 (57.1)	173 (89.2)	0.91 (0.45–1.85)
	**Malaria, all sources**^**c**^ **(*N*** **= 4027)**			**Confirmed malaria**^**d**^ **(*N*** **= 2812)**		
	**Cancer**^**e**^ **among malaria patients,** ***n*** **(%)**	**Cancer among comparators,** ***n*** **(%)**	**HR (95% CI)**	**Cancer**^**e**^ **among malaria patients,** ***n*** **(%)**	**Cancer among comparators,** ***n*** **(%)**	**HR (95% CI)**
All	202	3933	0.89 (0.77–1.02)	145	2559	0.98 (0.83–1.16)
Sex						
Male	112 (55.5)	2245 (57.1)	0.89 (0.74–1.07)	85 (58.6)	1522 (59.5)	1.00 (0.80–1.24)
Female	90 (44.6)	1688 (42.9)	0.89 (0.72–1.10)	60 (41.4)	1037 (40.5)	0.94 (0.73–1.23)
Follow-up time (y)						
< 5	44 (21.8)	947 (24.1)	0.72 (0.53–0.98)	27 (18.6)	498 (19.5)	0.70 (0.48–1.04)
5–9	50 (24.8)	944 (24.0)	0.77 (0.58–1.03)	36 (24.8)	605 (23.6)	0.82 (0.59–1.15)
10–19	81 (40.1)	1498 (38.1)	0.86 (0.69–1.08)	60 (41.4)	1077 (42.1)	0.86 (0.67–1.12)
20–30	27 (13.4)	544 (13.8)	0.96 (0.65–1.41)	22 (15.2)	379 (14.8)	1.17 (0.76–1.79)
Years of entry						
1987–1994	79 (39.1)	1510 (38.4)	0.99 (0.79–1.24)	58 (40.0)	985 (38.5)	1.16 (0.89–1.51)
1995–2004	101 (50.0)	1939 (49.3)	0.86 (0.70–1.05)	77 (53.1)	1331 (52.0)	0.94 (0.74–1.18)
2005–2015	22 (10.1)	484 (12.3)	0.72 (0.47–1.11)	10 (6.9)	243 (9.5)	0.62 (0.33–1.16)
Region of birth						
Sub-Saharan Africa	26 (12.9)	207 (5.3)	0.99 (0.66–1.50)	19 (13.1)	160 (6.3)	0.93 (0.58–1.49)
Non/low-endemic	176 (87.1)	3726 (94.7)	0.92 (0.79–1.07)	126 (86.9)	2399 (93.8)	1.05 (0.87–1.25)

*CI* confidence interval, *HR* hazard ratio, *N* total number, *n* number, *y* years

^a^Patients with malaria identified by all sources (Public Health Agency, microbiology and infectious disease departments, National Patient Register), without previous lymphoid neoplasms

^b^Patients with malaria identified by the Public Health Agency or microbiology and infectious disease departments, without previous lymphoid neoplasms

^c^Patients with malaria identified by all sources (Public Health Agency, microbiology and infectious disease departments, National Patient Register), without previous cancer

^d^Patients with malaria identified by the Public Health Agency or microbiology and infectious disease departments, without previous cancer

^e^All cancers except for non-melanoma skin cancers, leukaemia and lymphoma (ICD-7: 140-190, 192-199)

By categorising patients into four levels of exposure based on birth region and malaria diagnosis, we observed that malaria patients of endemic origin had a higher risk of lymphoid neoplasms compared to the population from non/low-endemic regions without previous malaria diagnosis (HR 2.36, 95% CI 1.05–5.33, adjHR 2.24, 95% CI 0.99–5.07), also when restricting the analysis to confirmed cases only (HR 2.94, 95% CI 1.29–6.67 and adjHR 2.88, 95% CI 1.27–6.54), while the risk for non-haematological cancer was rather decreased (HR 0.60, 95% CI 0.41–0.86, adjHR 0.61, 95% CI 0.42–0.90) (Additional file 1: Table S1).

SNOMED codes were available for all the lymphoid neoplasms among malaria cases and 98% of comparators. Histological classification showed that non-Hodgkin lymphoma was the most common type of lymphoid neoplasm among both malaria patients (63%) and in the comparator cohorts (72%). Two (10%) of the malaria patients with lymphoid neoplasms and 14 (5%) of the comparators had Hodgkin. Chronic lymphocytic leukaemia (CLL), one of the most common haematological disorder seen among Swedish controls (19%), was not as frequent among malaria patients (10%), and no cases were seen among malaria patients with endemic origin (Additional file 1: Table S2). The only case of Burkitt lymphoma was in an adult of endemic origin with a confirmed malaria diagnosis. There were additional variations in the proportion of NHL subgroups depending on the exposure and origin, but numbers were too small for comparative analysis. With the stricter lymphoma definition, a total of 12 malignant lymphomas were identified among malaria patients and 164 among comparators, but the risk was largely unchanged (HR 1.36, 95% CI 0.75–2.44).

In the subset of malaria patients with clinical data (*N* = 2009), we were able to assess disease severity, but none of the patients who later developed lymphoma had severe malaria according to WHO’s definition (WHO 2015). Among confirmed malaria cases, we also determined multiple episodes (including only new infections, not relapses or recrudescence) and species. One hundred seventy-nine of 2854 cases had two episodes and 25/2854 three or more additional episodes. However, none of these patients developed lymphoma. Among the 14 patients with confirmed malaria who later developed lymphoid neoplasms, most had been infected with *Plasmodium falciparum* (11/14), only one with *Plasmodium vivax* and two with *Plasmodium ovale.*

### Risk of other cancer

During follow-up, 202 non-haematological cancers were observed among the malaria cases and 3933 among the comparators, corresponding to a crude incidence rate of 3.65 (95% CI 3.18–4.19) cancers/1000 person-years among malaria patients and 4.25 (95% CI 4.12–4.39)/1000 person-years among malaria-free comparators. There was no significant difference in the overall risk for incident cancer for malaria patients, rather a tendency of decreased risk (HR 0.89, 95% CI 0.77–1.02), also after adjustment for sex, calendar period and birth region (adjHR 0.92, 95% CI 0.80–1.06) and after restricting the analysis to confirmed cases only (HR 0.98, 95% CI 0.83–1.16, adjHR 1.02, 95% CI 0.86–1.21) (Table 2).

We also investigated the distribution of cancer types according to malaria exposure and region of birth. The proportion of lymphoid neoplasms was markedly increased among malaria patients with Sub-Saharan origin (18%) compared to both comparators with the same origin (9%), as well as malaria patients (5%) and controls (6%) with non/low-endemic origin. Except for a high proportion of endocrinal tumours (mainly thyroid gland) and liver cancers among both malaria patients and comparators from Sub-Saharan Africa, no other major differences in the distribution of cancer types were observed (Additional file 1: Table S3).

### Sub-Saharan African cohort

In the population born in Sub-Saharan Africa (*N* = 171,756 individuals with 179,147 registered periods in Sweden), there was a total of 212 lymphoid neoplasms, six of these in 15,497 exposed person-years in 1305 individuals diagnosed with malaria in Sweden and 206 lymphoid neoplasms during 1,720,550 unexposed person-years in 170,451 individuals without previous malaria diagnosis, conferring to an event rate of 0.39 (95% CI 0.17–0.86) and 0.12 (95% CI 0.10–0.14), respectively (Table 3).

**Table 3:** Characteristics, incident lymphoid neoplasm and event rate in the population born in Sub-Saharan Africa (SSA) with and without malaria

	Population born in SSA, all registered periods of stay in Sweden from 1987					
	Periods after malaria diagnosis, *n* (%) (*N* = 1426)	Lymphoid neoplasms, *n* (%)	Event rate, *n* lymphoid neoplasms/1000 person-years (95% CI)	Periods without previous malaria diagnosis, *n* (%) (*N* = 177,721)	Lymphoid neoplasms, *n* (%)	Event rate, *n* lymphoid neoplasms/1000 person-years (95% CI)
**Total number of individuals**	1305	6	0.39 (0.17–0.86)	170451^a^	206	0.12 (0.10–0.14)
**Total follow-up time, person-years**	15,497			1,720,550		
**Sex**						
Male	976 (68.4)	4 (66.7)	0.39 (0.15–1.00)	94,286 (53.1)	125 (60.7)	0.14 (0.12–0.16)
Female	450 (31.6)	2 (33.3)	0.39 (0.10–1.55)	83,435 (47.0)	81 (39.3)	0.10 (0.08–0.12)
**Region of birth**						
East Africa	596 (41.8)	0	–	142,237 (80.0)	145 (70.4)	0.11 (0.09–0.13)
Middle Africa	166 (11.6)	2 (33.3)	1.17 (0.29–4.67)	8316 (4.7)	14 (6.8)	0.17 (0.10–0.30)
Western Africa	658 (46.1)	4 (66.7)	0.58 (0.22–1.56)	22,548 (12.7)	38 (18.5)	0.16 (0.12–0.22)
Southern Africa	6 (0.4)	0	–	4620 (2.6)	9 (4.4)	0.20 (0.11–0.39)
**Age at first immigration (y)**						
< 18	385 (27.0)	0	–	65,442 (36.8)	30 (14.6)	0.05 (0.03–0.07)
18–29	641 (45.0)	3 (50.0)	0.43 (0.14–1.33)	63,178 (35.6)	72 (35.0)	0.11 (0.09–0.14)
30–39	331 (23.2)	3 (50.0)	0.89 (0.29–2.77)	33,205 (18.7)	59 (28.6)	0.20 (0.15–0.25)
≥ 40	69 (4.8)	0	–	15,896 (8.9)	45 (21.8)	0.37 (0.28–0.50)
**Age at lymphoma/end of follow-up (y)**						
0–29	277 (19.4)	0	–	74,370 (41.9)	33 (16.0)	0.07 (0.05–0.10)
30–49	684 (48.0)	1 (16.7)	0.14 (0.02–0.10)	78,076 (43.9)	89 (43.2)	0.11 (0.09–0.13)
≥ 50	465 (32.6)	5 (83.3)	0.75 (0.31–1.81)	25,275 (14.2)	84 (40.8)	0.18 (0.15–0.23)
**Follow-up time from immigration (y)**						
< 5	232 (16.3)	0	–	67,420 (37.9)	74 (35.9)	0.37 (0.29–0.46)
5–9	252 (17.7)	0	–	51,952 (29.2)	37 (18.0)	0.10 (0.07–0.13)
10–19	431 (30.2)	2 (33.3)	0.44 (0.11–1.78)	32,085 (18.0)	51 (24.8)	0.11 (0.09–0.15)
20–30	511 (35.8)	4 (66.7)	0.45 (0.7–1.21)	26,264 (14.8)	44 (21.4)	0.06 (0.05–0.09)
**Total years in malaria-endemic country**						
0–9	165 (11.6)	0	–	31,747 (17.9)	18 (8.7)	0.05 (0.03–0.09)
10–19	268 (18.8)	0	–	40,642 (22.9)	16 (7.8)	0.04 (0.03–0.07)
20–29	582 (40.8)	3 (50.0)	0.48 (0.15–1.50)	55,915 (31.5)	67 (32.5)	0.11 (0.09–0.14)
≥ 30	411 (28.8)	3 (50.0)	0.73 (0.24–2.27)	49,417 (27.8)	105 (51.0)	0.24 (0.20–0.30)

Individuals included from the first immigration or from 1987 if immigration occurred before 1987

*CI* confidence interval, *N* total number, *n* number, *SSA* Sub-Saharan Africa, *y* year

^a^Includes 28 individuals that later become exposed

The risk of developing a lymphoid neoplasm was more than twice as high (HR 2.39, 95% CI 1.06–5.40) after a diagnosis of malaria, also after adjusting for sex and calendar period (adjHR 2.33, 95% CI 1.03–5.26) (Table 4). And when restricting the analysis to confirmed cases only, the association became stronger (HR 2.97, 95% CI 1.32–6.70, adjHR 2.88, 95% CI 1.27–6.51) (Table 4).

**Table 4:** Hazard ratios for incident lymphoid neoplasm and other cancer in malaria patients born in Sub-Saharan Africa (SSA)

Population born in SSA, all registered periods of stay in Sweden from 1987							
**Malaria all sources**^**a**^: **periods after malaria diagnosis *N* = 1426; periods without malaria diagnosis *N* = 177,721**					**Confirmed malaria**^**b**^**: periods after malaria diagnosis** ***N*** **= 1165;** **periods without malaria diagnosis** ***N* = 177,721**		
	**Lymphoid neoplasms in periods after malaria diagnosis,** ***n***	**Lymphoid neoplasms in periods without malaria diagnosis,** ***n***	HR (95% CI)	adjHR^c^ (95% CI)	**Lymphoid neoplasms in periods after malaria diagnosis,** ***n***	HR (95% CI)	adjHR^c^ (95% CI)
All	6	206	2.39 (1.06–5.40)	2.33 (1.03–5.26)	6	2.97 (1.32–6.70)	2.88 (1.27–6.51)
Time spent in an endemic region							
< 30 years	3	101	2.19 (0.69–6.94)	2.17 (0.68–6.90)	3	2.75 (0.87–8.74)	2.73 (0.86–8.70)
≥ 30 years	3	105	2.70 (0.85–8.51)	2.51 (0.79–7.94)	3	3.25 (1.03–10.28)	3.01 (0.95–9.56)
Sex							
Male	4	125	2.02 (0.74–5.47)	1.99 (0.73–5.41)	4	2.48 (0.91–6.72)	2.44 (0.90–6.63)
Female	2	81	3.12 (0.77–12.75)	3.36 (0.82–13.75)	2	3.96 (0.97–16.15)	4.31 (1.05–17.68)
**Malaria all sources**^**d**^**: periods after malaria diagnosis *N* = 1419; periods without malaria diagnosis *N* = 177,611**					**Confirmed malaria**^e^: **periods after malaria diagnosis** ***N*** **= 1160;** **periods without malaria diagnosis *N* = 177,611**		
	**Cancer**^**f**^ **in periods after malaria diagnosis,** ***n***	**Cancer in periods without malaria diagnosis,** ***n***	HR (95% CI)	adjHR^c^ (95% CI)	**Cancer**^**f**^ **in periods after malaria diagnosis,** ***n***	HR (95% CI)	adjHR^c^ (95% CI)
All	30	2376	1.01 (0.70–1.45)	1.14 (0.79–1.63)	22	0.92 (0.60–1.40)	1.04 (0.68–1.59)

*CI* confidence interval, *HR* hazard ratio, *adjHR* adjusted hazard ratio, *N* total number, *n* number, *SSA* Sub-Saharan Africa

^a^Patients with malaria identified by all sources (Public Health Agency, microbiology and infectious disease departments, National Patient Register), without previous lymphoid neoplasms

^b^Patients with malaria identified by the Public Health Agency or microbiology and infectious disease departments, without previous lymphoid neoplasms

^c^Adjusted for sex and calendar period. Age as an underlying timescale in all analyses

^d^Patients with malaria identified by all sources (Public Health Agency, microbiology and infectious disease departments, National Patient Register), without previous cancer

^e^Patients with malaria identified by the Public Health Agency or microbiology and infectious disease departments, without previous cancer

^f^All cancers except for non-melanoma skin cancers, leukaemia and lymphoma (ICD-7: 140-190, 192-199)

A tendency of stronger association among individuals with long attained time (≥ 30 years) in the endemic country as well as female sex was observed in stratified analyses (Table 4), but no interaction with neither total attained time (HR 3.18 for malaria diagnosis and time ≥ 30 years, 95% CI 1.01–10.03, *p* = 0.49) nor gender (HR 3.21 for malaria diagnosis and female sex, 95% CI 0.79–13.06, *p* = 0.10) could be demonstrated except for ≥ 30 years in confirmed cases of malaria (HR 3.18, 95% CI 1.01–10.03, *p* = 0.049).

After excluding individuals with a diagnosis of HIV or chronic hepatitis B or C before inclusion and censoring those diagnosed during follow-up, a total of 5 lymphoid neoplasms were observed among malaria patients and 154 among comparators. The overall risk for lymphoid neoplasms after malaria was still increased (HR 2.63, 95% CI 1.08–6.42), also after adjustments for sex and calendar period (HR 2.50, 95% CI 1.02–6.11) (Additional file 1: Table S4).

The risk for all-site non-haematological cancer did not differ significantly for malaria exposed vs unexposed (HR 1.01, 95% CI 0.70–1.45), independently of sex and calendar period (HR 1.14, 95% CI 0.79–1.63) (Table 4).

The malaria patients that developed lymphoid neoplasms all had confirmed malaria, were born in middle or western Africa, had immigrated to Sweden at 28–34 years of age and developed lymphoma after at least 10 years in Sweden. Four patients were diagnosed with lymphoma 5 to 13 years after the malaria diagnosis and the other two between 1 and 5 years after. Except one Hodgkin lymphoma and one myeloma, the remaining four (67%) were B cell-derived non-Hodgkin lymphoma, one of these a Burkitt lymphoma. Among the Sub-Saharan population without previous malaria diagnosed in Sweden, 119 (58%) of all lymphoid neoplasms were NHL (Additional file 1: Table S2). We observed a more pronounced risk in malaria patients when using the stricter definition of only malignant lymphoma (HR 3.49, 95% CI 1.42–8.56, adjHR 3.27, 95% CI 1.33–8.04), also when restricting the analysis to confirmed cases (HR 4.35, 95% CI 1.77–10.67, adjHR 4.05, 95% CI 1.65–9.96).

Among individuals without malaria diagnosis, 16% of those who developed a lymphoid neoplasm were children or young adults (< 30 years) at the time of diagnosis, and most lymphoid neoplasms were diagnosed at 30-49 years of age. Among malaria patients, the majority were diagnosed with lymphoma at > 50 years of age, and no one was diagnosed < 30 years of age (Table 3).

## Discussion

In this nationwide population-based cohort study of patients diagnosed with malaria in Sweden, we observed an over two times increased risk of incident lymphoid neoplasms in patients born in Sub-Saharan Africa, but no increased risk for patients with malaria born in Sweden. The risk was more pronounced for B cell lymphoma and in confirmed cases of malaria. No risk of non-haematological cancer was observed in malaria patients overall, and in patients from Sub-Saharan Africa, the risk was rather decreased.

### Comparisons with other studies

Previous studies from endemic areas have established an association between holoendemic *P. falciparum* and Burkitt lymphoma in African children, and a possible link with non-Burkitt lymphomas and cervical cancers in adults, but have been based on regional differences in malaria transmission and cancer incidence [6–8, 11], sero-epidemiological comparisons involving anti-malaria antibodies [26, 27] or Mendelian randomisation approach of sickle cell traits [28]. A nested case-control study from the UK found an association with previous malaria exposure in adults with sporadic Burkitt lymphoma; however, the definition of exposure included both treatment for malaria, prescribed chemoprophylaxis and a self-reported history of malaria, thus not microbiologically confirmed [12]. The few studies from non-endemic countries, suggesting an association between malaria and other lymphoid malignancies, lack longitudinal design and are prone to recall bias [13–15].

Here, we followed patients diagnosed with malaria for up to 30 years and found that individuals born in Sub-Saharan Africa had an increased risk of developing lymphoid neoplasms, 1.5–13 years after a confirmed malaria diagnosis. The long time frame makes reverse causation, i.e. higher probability of symptomatic malaria in individuals with an undiscovered lymphoma, unlikely. The risk was most pronounced for lymphomas discovered at > 50 years of age, indicating a prolonged effect and possibly different mechanisms compared to the endemic Burkitt lymphoma diagnosed in Sub-Saharan Africa which has a peak incidence at 6 years of age [29]. The only Burkitt lymphoma observed in our exposed population was a 52-year-old individual of endemic origin with confirmed *P. falciparum* and no known HIV or hepatitis.

### Interpretation

Infections play an important role in many cancers, attributing to at least 20% of all cancers globally [30]. Lymphoid neoplasms form a heterogenetic group both regarding morphology and aetiology. However, in particular, B cell-derived lymphomas have been linked to chronic inflammation and infections [31].

While the oncogenic role of Epstein-Barr virus (EBV) has been extensively studied, the mechanism linking *P. falciparum* to lymphoma development is still not completely understood. Recent studies have proposed that repeated malaria infections in early childhood leads to expansion and reactivation of latently EBV-infected B cells and deregulated expression of AID (activation-induced cytidine deaminase), inducing DNA damage enabling the *c-myc* translocation characterising Burkitt lymphoma [32, 33]. In murine models, infections with *Plasmodium* species have been observed to increase the incidence and promote the growth of various lymphoid neoplasms however also involving an oncogenic virus [34, 35]. Assessment of EBV and other oncoviruses as co-factors was not possible in our cohort since such serological markers were not available.

In this study, a single episode of malaria in individuals from non-endemic regions was not associated with an elevated risk of lymphoid neoplasms. Neither could we observe any additional risk in patients with severe malaria, which in our setting mostly affects non-immune travellers with first-time malaria [21]. Analogous to studies of endemic Burkitt, repeated or chronic malaria exposure is a more probable requirement for lymphomagenesis [36, 37], and possibly this could explain why the association with lymphoma was observed in malaria patients that were born and had spent over 20 years in an endemic region. Moreover, within the population born in Sub-Saharan Africa, being diagnosed with malaria in Sweden might reflect a higher level of previous exposure.

Patients with malaria may differ from the general population with respect to socioeconomic status, education, tobacco and alcohol use. However, there is no evidence that these risk factors have an important role in lymphoma development [31, 38]. In addition, the risk of solid tumours, including the proportion of lung cancers, was lower among malaria patients from endemic regions; thus, it is unlikely that our results are substantially confounded by these risk factors.

Sex was included in adjusted analyses since NHL is more common among men [39], and also a larger proportion of malaria patients were male, but interestingly stratified analysis in the Sub-Saharan African cohort showed a tendency of increased risk among women with malaria.

Since HIV and chronic hepatitis B were diagnosed more often among malaria patients and both these, as well as hepatitis C, have been associated with lymphoma [40–42], we did an additional analysis excluding or censoring individuals with HIV and hepatitis before or during follow-up. This did not substantially affect the risk for lymphoid neoplasms in malaria patients with an endemic origin. In Sweden, HIV and hepatitis are included in the pre-treatment screening of patients with haematological malignancies [43], and since HIV and hepatitis diagnosed up to 6 months after lymphoma were censored, it is unlikely that any additional diagnoses would have been missed among individuals with lymphoma.

Some autoimmune diseases such as rheumatoid arthritis have also been associated to lymphoma [44], but information on previous comorbidities was often lacking in immigrants, and there is no evidence suggesting that inflammatory diseases would be more common among immigrants from malaria-endemic areas.

### Limitations and strengths

This is to our knowledge the first published longitudinal study assessing the risk of incidental cancer in patients diagnosed with malaria. In addition to its nationwide population-based design with long-term follow-up, linkage with the Swedish national cancer register ensures unbiased and near-complete detection of the outcome. For confirmed malaria cases diagnosed from 1995, we also had clinical data enabling precise assessment of parasite species, disease severity and multiple episodes.

The setting in a malaria-free country where there is no risk of reinfection and malaria diagnoses are reported through compulsory notification aids correct classification of exposure.

However, misclassification of exposure, particularly among individuals with an endemic origin, cannot be entirely avoided since different degrees of exposure can have occurred before immigration or during later visits to endemic areas. Moreover, the persistence of undiagnosed asymptomatic infections after leaving the endemic setting can occur, as described in occasional case reports [45], but are likely to be rare. In addition, malaria transmission can vary greatly within a country as well as change over time, thus estimation of previous exposure based on country of birth will always be imprecise. We used the total number of years lived in an endemic region in an attempt to quantify previous exposure. However, even if the risk of lymphoma was somewhat increased in individuals with > 30 years stay in endemic areas and confirmed malaria, data indicates that being diagnosed with malaria in Sweden might be a more adequate marker of high exposure.

The Swedish surveillance system for communicable diseases has been known for high sensitivity [46], but to ensure complete coverage, we also used the National Patient Register. Despite only including patients treated in hospitals and at outpatient clinics that manage malaria, there will still have been some degree of misdiagnosing, explaining the stronger association observed among confirmed cases.

The large proportion of immigrants resulted in partly incomplete follow-up due to frequent emigration, which we attempted to optimise by including subsequent re-immigration periods in the Sub-Saharan African cohort. This could potentially introduce a detection bias if patients diagnosed with malaria have a different migration pattern compared to the controls. Likewise, a presumably more active health care-seeking behaviour among immigrants diagnosed with malaria in Sweden would imply a higher probability of being diagnosed with other diseases such as lymphoma. However, both these potential biases would also have affected the incidence of all-site cancers, which we did not observe. Rather, there was a tendency of an overall lower cancer incidence in the Sub-Saharan African cohort, possibly due to selection of healthier individuals among immigrants (healthy migrant effect) or the return of migrants to their home country due to disease (salmon effect).

### Implications

Our results indicate the need for a raised awareness among clinicians to consider lymphomas in malaria patients from endemic regions, many years after exposure. However, the study does not provide evidence for a causal link between malaria and lymphoid neoplasms, nor is it designed to understand the underlying mechanisms for the association observed. A larger cohort of malaria patients would be required to confirm our observations, preferably both with a similar setup in a non-endemic country and in regions of varying malaria endemicity.

## Conclusions

A single episode of malaria in a traveller from a non-endemic country is not associated with an increased risk for lymphoid neoplasms. However, individuals that have grown up in Sub-Saharan Africa and diagnosed with malaria in Sweden have an increased risk for lymphoid neoplasms, in particular, B cell-derived lymphomas, several years after malaria diagnosis. There was no increased risk for non-haematological cancers in malaria patients independent of origin. The results suggest a possible association between repeated episodes of malaria during childhood and development of lymphoid neoplasm during adulthood.

## Supplementary information

**Additional file 1: Table S1.** Crude and adjusted Hazard Ratio (HR) for incident lymphoid neoplasm and all-site cancer according to malaria exposure and region of birth. **Table S2.** Subtypes of lymphoid neoplasms in patients with malaria and comparators, according to region of birth. **Table S3** Types of cancers (all) in patients with malaria and comparators, according to region of birth. **Table S4.** Crude and adjusted HR for incident lymphoid neoplasm, in population from Sub-Saharan Africa (SSA) without previous HIV and Chronic Hepatitis.

## Data Availability

The data used in this study derives from the National Surveillance Database at the Public Health Agency of Sweden, the National Patient Register and the Cancer Register at the National Board of Health and Welfare in Sweden and the Total Population Register at Statistics of Sweden. The data contains sensitive personal information, and according to the Swedish law and the General Data Protection Regulation, the authors are not permitted to share the datasets used in this study with third parties. Details on the application procedures for data usage are available on the homepages of the respective registries. For statistical coding related to data analysis, contact the corresponding author at katja.wyss@sll.se.

## Abbreviations

ALL: Acute lymphoblastic leukaemia
CI: Confidence interval
CLL: Chronic lymphatic leukaemia
EBV: Epstein-Barr virus
HR: Hazard ratio
adjHR: Adjusted hazard ratio
ICD: International Classification of Disease
InterLymph: International Lymphoma Epidemiology Consortium
NHL: Non-Hodgkin lymphoma
NPR: National Patient Register
PIN: Personal identification number
SNOMED: Systematized Nomenclature of Medicine
SSA: Sub-Saharan Africa
TPR: Total Population Register
