# Supplementary material for: Malaria and risk of lymphoid neoplasms and other cancer: a nationwide population-based cohort study
Source: BMC Med. 2020 Oct 30;18:296. doi: 10.1186/s12916-020-01759-8 (PMC7596993; doi:10.1186/s12916-020-01759-8)
Supplement: Supplementary file 1 — Additional file 1: Table S1. Crude and adjusted Hazard Ratio (HR) for incident lymphoid neoplasm and all-site cancer according to malaria exposure and region of birth. Table S2. Subtypes of lymphoid neoplasms in patients with malaria and comparators, according to region of birth. Table S3 Types of cancers (all) in patients with malaria and comparators, according to region of birth. Table S4. Crude and adjusted HR for incident lymphoid neoplasm, in population from Sub-Saharan Africa (SSA) without previous HIV and Chronic Hepatitis. [file 12916_2020_1759_MOESM1_ESM.docx]

**Additional file 1: Supplementary tables**

**Table S1.** Crude and adjusted Hazard Ratio (HR) for incident lymphoid neoplasm and other cancer according to malaria exposure and region of birth.

|  | Malaria all sources, without previous lymphoid neoplasm  N = 4125 | | | Confirmed malaria, without previous lymphoid neoplasm  N=2854 | | |
| --- | --- | --- | --- | --- | --- | --- |
|  | **Lymphoid neoplasms, n** | **HR (95% CI)** | **adjHR^b^ (95% CI)** | **Lymphoid neoplasms, n** | **HR (95% CI)** | **adjHR^b^ (95% CI)** |
| Comparators, non-endemic origin | 279 | 1 | 1 | 173 | 1 | 1 |
| Malaria patients, non-endemic origin | 14 | 1.05 (0.62-1.80) | 1.05 (0.61-1.79) | 8 | 0.91 (0.45-1.85) | 0.91 (0.45-1.85) |
| Comparators, endemic origin^a^ | 25 | 1.29 (0.84-1.96) | 1.24 (0.81-1.89) | 21 | 1.34 (0.84-2.14) | 1.30 (0.81-2.07) |
| Malaria cases, endemic origin | 6 | 2.36 (1.05-5.33) | 2.24 (0.99-5.07) | 6 | 2.94 (1.29-6.67) | 2.88 (1.27-6.54) |
|  | **Malaria all sources, without previous cancers**  **N=4 027** | | | **Confirmed malaria, without previous cancers**  **N=2 812** | | |
|  | **Cancer^c^, n** | **HR (95% CI)** | **adjHR^2^ (95% CI)** | **Cancer^c^, n** | **HR (95% CI)** | **adjHR^2^ (95% CI)** |
| Comparators, non-endemic origin | 3726 | 1 | 1 | 2399 | 1 | 1 |
| Malaria patients, non-endemic origin | 176 | 0.92 (0.79-1.07) | 0.91 (0.78-1.06) | 126 | 1.05 (0.87-1.25) | 1.04 (0.87-1.24) |
| Comparators, endemic origin | 207 | 0.60 (0.52-0.69) | 0.62 (0.54-0.71) | 160 | 0.59 (0.50-0.70) | 0.62 (0.52-0.73) |
| Malaria patients, endemic origin | 26 | 0.60 (0.41-0.86) | 0.61 (0.42-0.90) | 19 | 0.56 (0.36-0.88) | 0.57 (0.36-0.89) |

Abbreviations: CI confidence interval, HR hazard ratio, N total number, n number, y years

^a^ Endemic/non-endemic origin -individuals born in countries within Eastern Africa, Western Africa, Middle Africa, Southern Africa were classified as of endemic origin, all other as of non/low-endemic origin.

^b^ Adjusted for sex and calendar period with age as underlying time-scale.

^c^ All cancers except non-melanoma skin cancers, leukemia, lymphoid neoplasms according to ICD-7: 140-190,192-199.

**Table S2** Subtypes of lymphoid neoplasms in patients with malaria and comparators, according to region of birth.

| Subtypes^a^ | Malaria patients, non-endemic origin  n (%) | Matched comparators, non-endemic origin  n (%) | Malaria patients, endemic origin  n (%) | Matched comparators, endemic origin  n (%) | Sub-Sahara African cohort without malaria^b^  n(%) |
| --- | --- | --- | --- | --- | --- |
| All | 14 | 279 | 6 | 25 | 206 |
| NHL, n (%) | **8 (57)** | **200 (72)** | **4 (67)** | **15 (60)** | **119 (58)** |
| DLBCL | 1 (7) | 46 (16) | 1 (17) | 2 (8) | 24 (12) |
| Lymphoplasmacytic lymphoma | 1 (7) | 10 (4) | 1 (17) | 1 (4) | 3 (1) |
| CLL | 2 (14) | 52 (19) | 0 | 2 (8) | 18 (9) |
| Burkitts | 0 | 0 | 1 (17) | 0 | 8 (4) |
| Follicular lymphoma /MALT | 1 (7) | 27 (10) | 1 (17) | 3 (12) | 16 (8) |
| B-cell other | 1 (7) | 24 (9) | 0 | 1 (4) | 5 (2) |
| T-cell lymphoma | 1 (7) | 16 (6) | 0 | 3 (12) | 10 (5) |
| ALL (B/T cell) | 0 | 11 (4) | 0 | 0 | 13 (6) |
| NHL, unspecified | 1 (7) | 14 (5) | 0 | 3 (12) | 22 (11) |
| Hodgkin lymphoma, n (%) | 1 (7) | 12 (4) | 1 (17) | 2 (8) | 39 (19) |
| Myeloma, n (%) | 4 (29) | 54 (19) | 1 (17) | 7 (28) | 42 (20) |
| Lymphoproliferative disorders, n (%) | 1 (7) | 7 (3) | 0 | 0 | 2 (1) |
| Uncategorized, n (%) | 0 | 6 (2) | 0 | 1 (4) | 4 (2) |

Abbreviations: NHL= Non Hodgkin lymphoma, DLBCL = Diffuse large B-cell lymphoma, CLL=Chronic lymphocytic leukemia, MALT= Marginal zone lymphoma, B-cell other includes Mantle cell lymphoma and Hairy cell leukemia as well as unspecified B-cell lymphomas, ALL= Acute lymphoblastic leukemia, n=number

^a^ Subtypes categorized using SNOMED 2 codes (Systematized Nomenclature of Medicine codes available 1993-2015). 7 unexposed individuals could not be categorized because of missing (n=1) or incorrect (n=6) SNOMED2 codes in the matched population and 4 missing SNOMED2 codes in the Sub-Saharan African Population.

^b^ Unmatched comparator cohort constituted of all individuals registered in Sweden 1987-2015 with birth region in Sub-Saharan Africa and without previous malaria diagnosed in Sweden.

**Table S3** Types of cancers (all) in patients with malaria and comparators, according to region of birth.

| **Type of cancer** | **Malaria patients,**  **non-endemic origin**  **n (%)** | **Matched comparators, non-endemic origin**  **n (%)** | **Malaria patients, endemic origin**  **n (%)** | **Matched comparators,**  **endemic origin**  **n (%)** | **Sub-Sahara African cohort without malaria^a^**  **n (%)** |
| --- | --- | --- | --- | --- | --- |
| **All** | 190 | 3976 | 33 | 237 | 2668 |
| **Oropharynx** | 3 (1.6) | 75 (1.9) | 0 | 5 (2.1) | 52 (1.9) |
| **Stomach** | 1 (0.5) | 62 (1.6) | 1 (3.0) | 5 (2.1) | 37 (1.4) |
| **Liver** | 2 (1.1) | 63 (1.6) | 1 (3.0) | 12 (5.1) | 121 (4.5) |
| **Colorectal** | 19 (10.0) | 407 (10.2) | 3 (9.1) | 17 (7.2) | 196 (7.3) |
| **Gastrointestinal** | 5 (2.5) | 136 (3.4) | 1 (2.7) | 8 (3.3) | 87 (3.3) |
| **Respiratory** | 9 (4.7) | 259 (6.5) | 2 (6.1) | 17 (7.2) | 127 (4.8) |
| **Mammae** | 19 (10.0) | 380 (9.6) | 3 (9.1) | 17 (7.2) | 347 (13.0) |
| **Female genitals** | 30 (15.8) | 605 (15.2) | 6 (18.2) | 28 (11.8) | 637 (23.9) |
| **Male genitals** | 50 (26.3) | 868 (21.8) | 5 (15.1) | 37 (15.6) | 238 (18.9) |
| **Urinary tract** | 8 (4.2) | 234 (5.9) | 0 | 11 (4.6) | 101 (3.8) |
| **Melanoma** | 17 (9.0) | 289 (7.3) | 1 (3.0) | 3 (1.3) | 26 (1.0) |
| **CNS +eye** | 1 (0.5) | 121 (3.0) | 0 (0) | 10 (4.2) | 113 (4.2) |
| **Endocrinal** | 6 (3.2) | 84 (2.1) | 2 (6.1) | 30 (12.7) | 219 (8.2) |
| **Bone +soft tissue** | 0 | 36 (0.9) | 1 (3.0) | 4 (1.7) | 41 (1.5) |
| **Lymphoma** | 9 (4.7) | 218 (5.5) | 6 (18.2) | 22 (9.3) | 205 (7.7) |
| **Leukemia** | 5 (2.6) | 44 (1.1) | 0 | 5 (2.1) | 69 (2.6) |
| **Myelofibrosis /PCV** | 0 | 21 (0.5) | 1 (3.0) | 3 (1.3) | 18 (0.7) |
| **Unknown primary** | 6 (3.2) | 75 (1.9) | 0 | 4 (1.7) | 34 (1.3) |

Abbreviations: CNS= Central Nervous System, PCV= Polycythemia Vera, n=number

All cancers including leukemia and lymphoid neoplasms (but not non-melanoma skin cancers), according to ICD-7: 140-190,192-207.

^a^ Unmatched comparator cohort constituted of all individuals registered in Sweden 1987-2015 with birth region in Sub-Saharan Africa and without previous malaria diagnosed in Sweden.

**Table S4.** Crude and adjusted HR for incident lymphoid neoplasm, in population from Sub-Saharan Africa (SSA) without previous HIV and Chronic Hepatitis.^a^

| Population born in SSA. Malaria all sources, without HIV/Hepatitis | | | |
| --- | --- | --- | --- |
|  | **Lymphoid neoplasm, n** | **HR (95% CI)** | **adjHR^b^ (95% CI)** |
| Periods without malaria  (N=177 087) | 154 | 1 | 1 |
| Periods after malaria  (N=1375) | 5 | 2.63 (1.08-6.42) | 2.50 (1.02-6.11) |

Abbreviations: CI = confidence interval, HR = hazard ratio, adjHR = adjusted hazard ratio, n = number, N =total number, SSA = Sub-Saharan Africa

^a^ Individuals with HIV or Chronic Hepatitis diagnose prior to inclusion excluded. Cases and controls with HIV or Chronic Hepatitis diagnose during follow up or within 6 months after lymphoma diagnose were censored.

^b^ Adjusted for sex and calendar period. Age as underlying time-scale in all analyses.

Excluded because of HIV/Hepatitis diagnose before inclusion: Malaria exposed: 1 with Hepatitis B and 7 with HIV. Unexposed: 357 with HIV, 249 with Hepatitis B, 46 with Hepatitis C. 9 with Hepatitis B +HIV. 6 with Hepatitis C +HIV. 10 with Hepatitis B +Hepatitis C. = 685

Censored during follow up because of HIV/Hepatitis diagnose: Malaria exposed with HIV: 42 (one with later lymphoma), exposed with Hepatitis B: 34. Exposed with Hepatitis C: 6. Exposed with HIV +Hepatitis B: 3. Exposed with HIV +Hepatitis C: 1. Unexposed with HIV: 2,186. Unexposed with Hepatitis B: 3,892. Unexposed with Hepatitis C: 697. Unexposed with HIV +Hepatitis B: 34. Unexposed with HIV and Hepatitis C: 3. Unexposed with Hepatitis B +Hepatitis C: 39.

43 malaria cases reclassified as unexposed because of HV/Chronic Hepatitis diagnose before malaria diagnose.

Censored because of HIV or Hepatitis diagnose within 6 months after lymphoma diagnose: 4 malaria exposed with HIV, 2 unexposed with Hepatitis B, 1 unexposed with Hepatitis C. (1 exposed with Hepatitis B within 6 m after lymphoma but this one already censored because of HIV before lymphoma)
